# Link Between Topographic Memory and the Combined Presentation of ADHD (ADHD-C): A Pilot Study

**DOI:** 10.3389/fpsyt.2021.647243

**Published:** 2021-06-17

**Authors:** Noemi Faedda, Cecilia Guariglia, Laura Piccardi, Giulia Natalucci, Serena Rossetti, Valentina Baglioni, Danilo Alunni Fegatelli, Maria Romani, Miriam Vigliante, Vincenzo Guidetti

**Affiliations:** ^1^Section of Child and Adolescents Neuropsychiatry, Department of Human Neuroscience, Sapienza University of Rome, Rome, Italy; ^2^Department of Psychology, Sapienza University of Rome, Rome, Italy; ^3^Cognitive and Motor Rehabilitation and Neuroimaging Unit, Scientific Institute for Research, Hospitalization and Healthcare (IRCCS) Fondazione Santa Lucia, Rome, Italy; ^4^Department of Dynamic and Clinical Psychology, Sapienza University of Rome, Rome, Italy; ^5^Department of Public Health and Infectious Diseases, Sapienza University of Rome, Rome, Italy

**Keywords:** navigational memory, executive function, attention deficit hyperactivity disorder, navigational skills, topographic memory

## Abstract

**Background:** Topographic memory is the ability to reach various places by recognizing spatial layouts and getting oriented in familiar environments. It involves several different cognitive abilities, in particular executive functions (EF), such as attention, working memory, and planning. Children with attention deficit hyperactivity disorder (ADHD) show impairments in inhibitory control, regulation of attention, planning, and working memory.

**Aim:** The aim of this study was to evaluate the topographic memory in children with ADHD-combined subtype (ADHD-C).

**Method:** Fifteen children (8–10 years) with a diagnosis of ADHD-C (DSM-5) (ADHD-C group) were compared to 15 children with typical development (TD group) of the same age. All children performed Raven's colored progressive matrices (CPM) test to obtain a measure related with cognitive functioning. The walking Corsi test (WalCT), a large-scale version of the Corsi block-tapping test, was used to assess topographic memory in experimental environment.

**Results:** A higher impairment was observed in ADHD-C than TD with significant differences in the WalCT, in particular on the topographic short-term memory (TSTM) task, on the topographic learning (TL) task, and on the repetition number (RN) task during the TL task. Perseverative errors were reported in performing the square-sequence in the WalCT. Zero-order correlations showed a positive correlation between TSTM and auditory attention, and memory of design of NEPSY-II and digit span of WISC-IV. No statistically significant differences were found between the ADHD-C group and TD group in the TL task in the WalCT condition.

**Conclusion:** In ADHD-C, initial topographic learning was compromised whereas the long-term retention of learned topographical material seemed to not be impaired. In particular, these impairments seem to be linked with difficulties in sustained attention, in spatial memory for novel visual materials, in a poor working memory, and in perseverative behaviors.

## Introduction

### Development of Topographic Memory and Spatial Cognition

Human spatial cognition is a fundamental ability in humans. The visual and tactile world consists of objects situated in space, so it is essential from the first months of life to understand the characteristics of the surrounding area and object positions in it. This knowledge allows children to mentally and physically organize objects in their world. Indeed spatial awareness and spatial relations allow children to locate objects and navigate successfully in their environment, while spatial language allows children to express specific needs and describe the world (1).

Topographic memory is one component of spatial cognition that corresponds to the ability to reach various places in the environment, recognize spatial layouts, orient in familiar environments (2, 3), and to encode and maintain online sequences of environmental cues that are central during navigation (4, 5).

Topographic memory involves several cognitive processes and skills such as the ability to retain the spatial layout of an environment, to memorize and recognize complex visuo-spatial configurations, to find a shortcut between two locations, or to create an interconnected network among different paths (6–8). For a successful topographic memory, individuals have to access at least two types of spatial representations: the online representation of their position in the environment, and the offline representation of the environment (9). Therefore, it is essential that the individual create a stable mental representation of the environment, representing a “cognitive map” (7, 10, 11).

Indeed, human navigation develops gradually at distinct time points (12, 13), in parallel with the development of executive functions (EF) and language (5). By the age of 6–9 months, children are just able to use path integration, that is the ability to memorize their movements in the environment, and to get oriented on the basis of geometric features of the environment. They become able to deal with a spatial array from a novel viewpoint using landmarks only at 5 years old (5). Cognitive mapping starts to develop very late, at 7–8 years of age, and is fully functional by the age of 10 (12). However, the accuracy and the efficiency of navigational abilities continues to improve into adolescence, depending, at least in part, on the maturation of sensory and motor systems, combined with environmental feedback. In order to navigate in a successful way, humans must be able to process, integrate, and manipulate information derived from internal and external factors pertaining to time and space. The body's sensations and perceptions, environmental signals, and landmarks or reference points, are equally important for reaching a place and memorizing a path (13).

### Links Between Spatial Navigation and ADHD

Successful navigation is known to be strictly connected with the activity of several brain areas such as parieto-medial temporal networks (14), the posterior cingulate cortex (PCC), the retrosplenial cortex (RSC) (15), and the medial temporal lobe structures. Several neuroimaging studies have also revealed an increased activity in prefrontal areas during spatial navigation tasks (16–20). As described above, human navigation is constituted by multicomponent abilities, which need several cognitive processes, such as attention, memory, perception, and decision-making, for correct development and functioning. Indeed, even a single impairment in these processes may negatively affect navigation (21–24). Recently, in the adult population, several variable levels of navigational skills were reported and a specific developmental disorder, developmental topographical disorientation (DTD), was described. This disorder is characterized by a life-long inability in orientation despite otherwise well-preserved cognitive functions, and without other neurological conditions (25–31). This suggests the importance of understanding if and how the development of navigational skills is affected by the development of the other cognitive domains, particularly executive functions with specific attention toward working memory that is known to be important in maintaining online navigational information.

In this regard, the investigation of the spatial navigation in children, presenting this selective impairment, may shed light on those factors causative of the development of navigational skills. For this purpose, the study of topographic memory in children with attention deficit hyperactivity disorder (ADHD) could be crucial as a disorder of EFs, which are proposed to be a core deficit of this pathology (32, 33). Willcutt et al. (34) conducted a meta-analysis to evaluate the performance in executive function tasks in children and adolescents with ADHD showing weaknesses in several key EF domains, but the strongest and most consistent effects were obtained on measures of response inhibition, vigilance, spatial working memory, and some measures of planning. One of the most prominent cognitive weaknesses in patients with ADHD appears to be visuospatial working memory (VSWM), including short-term memory (STM) and central executive (CE) function (35). ADHD-combined subtype (ADHD-C), described as the more pervasive and impairing form of the disorder (36), shows significantly higher impairing rates in the functioning of vigilance, sustained attention, visual attention task, especially inhibition and shifting, visuo-spatial short-term memory, working memory, short-term memory compared to TD, and the other ADHD subtypes (37–41). Pasini et al. (42) conducted a study on attention and executive functions profile in drug-naive ADHD subtypes, founding that ADHD patients, inattentive and combined subtypes, differ from controls on response inhibition, divided attention, phonological, and visual object working memory and on variability of reaction times (43).

Attention and executive functions (EF), in particular inhibition and VSWM, play a critical role in the development of topographic memory, so it is important to investigate the development of topographic memory in children with ADHD that tend to fail in tasks both requiring an attentional load and involving EF (44–46). Furthermore, deficits in *executive functions* could also affect learning processes. In particular, several studies and clinical evidence (47–50) showed that children with ADHD present an impairment in sustained attention, flexibility, problem solving, and lower levels of task persistence, which has an impact on learning processes.

To investigate the development of navigational skills in ADHD children, it is important to consider that the age period of 8–10 years represents a crucial developmental phase for both executive functions and navigational skills [e.g., (4, 51–54)]. Previous studies found that the abilities to bind geometric environmental features, landmark identity, and directional estimations get developed at the age of 6–8 years, in accordance with the development of visuospatial processing and language (5, 7). Egocentric information, which includes spatial information about the location of the subject in the environment, is gradually supplemented with allocentric coding, which involves spatial information about the reciprocal object's positions (55). Furthermore, the relation-place strategies required for cognitive mapping start to develop around 7–8 years of age and are fully functioning by the age of 10 (56–60). It is important to note that several previous studies showed a cortical maturation delay in ADHD children, in particular in the prefrontal regions involved in attention, planning, and navigational abilities [e.g., (61–64)].

## Aim and Hypothesis

The aim of this study was to evaluate the topographic memory in children with ADHD-C compared with a group of typical developmental (TD) children. To this purpose, we used the WalCT (4, 51, 65, 66), a test widely used both in clinical and experimental settings to study topographic memory. This study was novel in its attempt to compare the performance of children with ADHD-C with a control group without any ADHD symptoms on topographical memory. The aim was to address the following questions:

1. Does the ADHD-C group perform poorly on the topographic short-term memory task compared to TD children?
  Based on previous studies that confirmed a strong relationship between working memory and navigation (4, 67–74) and based on previous results showing low working-memory performances in children with ADHD [e.g., (75)], poor performance on the topographic short-term memory (TSTM) task in children with ADHD-C compared to TD children was expected. To support of this hypothesis, Kofler et al. (76) found that impaired working memory in children with ADHD determined consistent difficulties in anticipating, planning, enacting, and maintaining goal-directed actions, all fundamental abilities for successful navigation.
2. Does the ADHD-C group perform poorly on topographic learning task compared to TD children?
  To our knowledge, this study is the first that assesses, in children with ADHD-C, not only some aspects of topographic short-term memory, but also the ability to learn new paths. It is known that sustained attention and flexibility in problem solving play an important role in learning and that these abilities seem to be compromised in children with ADHD (47, 49, 50). For such a reason, we also expected to find deficits in learning paths in children with ADHD-C but not in children with TD.
3. Does the ADHD-C group perform poorly on the topographic delayed recall (TDR) task compared to TD children?
  Few studies have assessed long-term memory (both learning and delayed recall) in children with ADHD. A recent meta-analysis (77) found that adults with ADHD performed significantly worse than controls on verbal but not on visual long-term memory and memory acquisition subtests. A long-term memory deficit was strongly statistically related to memory acquisition deficits. In contrast, no retrieval problems were observable suggesting that memory deficits in adults with ADHD reflect a learning deficit induced at the stage of encoding. Furthermore, Skowronek (78) showed equal or enhanced performance on long-term episodic tasks in children with ADHD compared to TD children. Based on this evidence, we hypothesize that we will not find any differences in long-term memory between the two groups.
4. Are there any correlations between topographic memory and executive function performances in the ADHD-C group?
  Topographic memory involves several different cognitive abilities, in particular executive functions (EFs), such as attention and working memory (21, 22, 24). To date no studies have evaluated the correlations between topographic memory and executive function performances in ADHD-C children, we expected a positive correlation between topographic short-term memory (TSTM) and the “attention and executive function” domain of NEPSY-II and topographic short-term memory (TSTM) and the working memory index of WISC-IV.

It is important to note that some navigational tasks might be impaired in both groups (children with ADHD and TD) because of age/stage of development. In particular, we considered the ability to use cognitive maps. Indeed, Lehnung et al. (56) showed that 10-year-old children tended to use cognitive-map strategies at greater rates and generally made fewer errors than younger children in a spatial orientation task, furthermore children who suffered from a traumatic brain injury before 10 years of age showed greater long-term impairments in the ability to orient themselves by means of cognitive maps. Newcombe (13) reported that by around 12 years, we can observe adult-level performance and adult patterns of individual differences on cognitive mapping tasks and these abilities continue to improve, along with the adaptive combination of various kinds of input, at least until adolescence. Each of these lines of development depends at least in part on the maturation of sensory and motor systems, combined with environmental feedback. Taken together, these findings suggest that several complex spatial cognition abilities develop rapidly between 7 and 12 years of age (79). Furthermore, different complex navigational skills depend on the development and maturation of several networks and brain areas such as the hippocampus, retrosplenial cortex, and prefrontal cortex (PFC) whose development is prolonged, continuing into adolescence [e.g., (15, 51, 80)].

In addition, the relation-place strategies required for cognitive mapping start to develop around 7 or 8 years of age and are fully functional by the age of 10 (56–60). Therefore, both groups, with ADHD-C and TD children, might have difficulties in transforming egocentric information into an allocentric representation of the experimental setting.

## Materials and Methods

### Participants

For this study, we assessed a sample of 100 children and adolescents (aged between 5.1 and 15.4 years), that had been referred to the Department of Human Neuroscience with a diagnostic suspicion of ADHD by parents, teachers, or a pediatrician. All children and parents underwent an initial interview with a child neuropsychiatrist who recorded the child's medical history, followed by an evaluation performed by a multidisciplinary team of experts constituted by a neuropsychiatrist and four psychologists. For the diagnosis, several neuropsychological and emotional tests were administered (see Supplementary Material).

After this assessment, for the ADHD-C group, patients needed to meet the following criteria for hyperkinetic disorder (HKD) for ICD-10 (81) and ADHD-C (48): six (or more) symptoms of hyperactivity and impulsivity and six (or more) symptoms of inattention persisting for at least 6 months to a degree that is inconsistent with developmental level and that negatively impacts social and academic/occupational activities. We chose to focus on the ADHD-C subtype because it defines a more pervasive and generally more impairing form of the disorder (36). In particular, some studies highlighted an association between specific subtypes of ADHD and TD and specific executive function impairments (38–40). In particular, children and adolescents with the ADHD-C subtype seemed to show significantly more impaired functioning on perseverative errors, visual attention tasks, especially inhibition and shifting, visuo-spatial short-term memory, working memory, and shortterm memory compared to TD and the other ADHD subtypes (38–41, 82). Furthermore, besides having a diagnosis of ADHD-C, the individuals included in the study had to meet the following criteria of inclusion: age 8–10, I.Q. ≥85, an average score on Raven's colored progressive matrices (percentile rank ≥ 37.5 = category average) (83), no previous treatment, no other diagnosis of a comorbidity, no primary visual or hearing impairments, and no other neurological or organic disease. After they were initially screened to determine their eligibility based on the aforementioned criteria, a sample of 15 children (age range: 8–10 y, M= 8.73, s.d.: 0.70; M:12; F:3) with a diagnosis of ADHD-C (experimental group: EG), based on DSM-5 (48) were recruited from the Department of Human Neuroscience.

We conducted an informal interview with parents of children that took part in the study as the control group (TD group) before the enrolment screening. None of these children had ever been previously reported for emotional, behavioral, or learning difficulties. No primary visual and hearing impairments, neurological and organic disease, or other neurodevelopmental issues were described. The individuals included in the study as TD had to meet the following criteria of inclusion: age 8–10, an average score on Raven's colored progressive matrices (percentile rank ≥ 37.5 = category average) (83), no emotional, behavioral, or learning difficulties, no primary visual or hearing impairments, no other neurodevelopmental, neurological, or organic disease. A typical developmental (TD) sample of 15 children (age range: 8–10 y, M = 8.87, s.d.: 0.83; M:10 F:5), with an average score on Raven's colored progressive matrices (percentile rank ≥ 37.5 = category average) [see Table 1; (84, 85)] without ADHD took part in the study as the comparison group (TD group).

**Table 1:** Descriptive statistics of the sample.

	**ADHD-C**	**TD**
Boys	12	10
Girls	3	5
Age	8.73 (M)	8.87 (M)
Raven	24.67	27.67
SES	Class 2: household income	Class 2: household income

We administered Raven's colored progressive matrices to both groups to obtain a measure of non-verbal intelligence and to exclude the presence of a visuospatial deficit. All subjects were of Caucasian origin and came from families with middle-class socio economic status (class 2: household income = €28,000–55,000; current Italian economic parameters), confirmed during the interview with parents. TD children were recruited from primary schools, describing the study protocol individually to their parents. Table 1 reports the descriptive statistics for the sample.

An informed written consent form, requiring active consent from caregivers, was signed. Each child, before taking part in the study, give an informed verbal assent. This research has been revised and approved by the Local Ethics Committees (Protocol number:416/16) in accordance with the tenets of the latest Declaration of Helsinki.

### Instruments

#### Neuropsychological Testing Administered to ADHD and TD Children

##### Raven's Colored Progressive Matrices (CPM)

All children in both the groups performed Raven's CPM to obtain a cognitive functioning measurement. The CPM is internationally recognized as a culture-fair test of non-verbal intelligence (84, 85) designed for the use of children between the ages of 5½ and 11½. The test consists of 36 items in three sets (A, Ab, B), with 12 items per set (86). As reported in Table 1, the ADHD-C group and TD group fell in the average range (ADHD-C group, raw score = 24.67, TD group, raw score = 27.67).

##### The Walking Corsi Test (WalCT)

**Figure 1:**
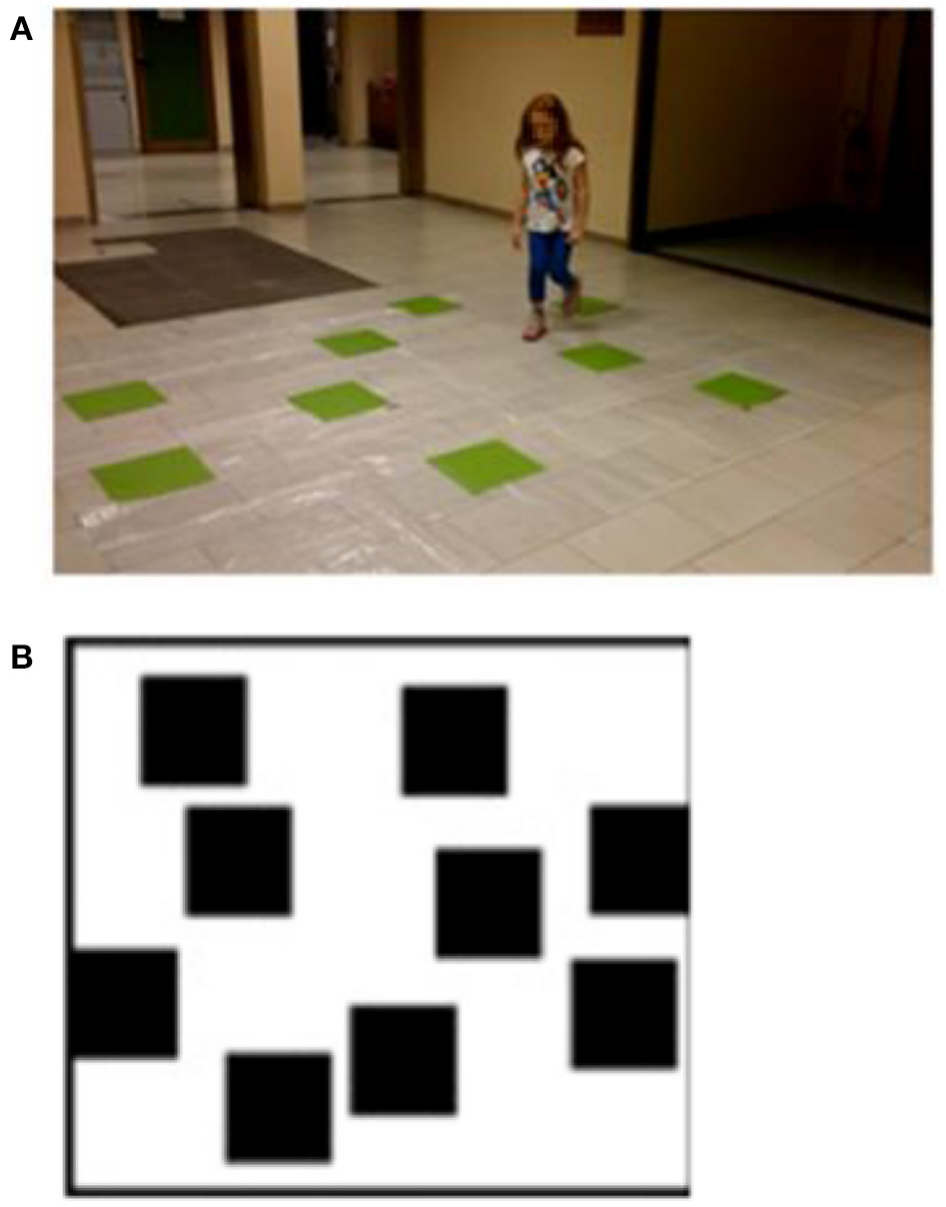
**(A)** Walking Corsi test (WalCT). The WalCT was the apparatus used for measuring topographical memory. Nine black squares (30 × 30 cm) were placed on a 2.50 × 3.00 m white carpet. The scaled position and the relative spatial layout of the squares were the same as in the Corsi block-tapping test (CBT). **(B)** The outline map of the WalCT on which children had to draw the previously learned path. Written informed consent was obtained from the parents of the child represented in the figure for publication of this experiment. A copy of the written consent is available for review by the Editor-in-Chief of this journal.

The WalCT (4, 5, 51, 65, 66) (Figure 1), a large-scale version of the Corsi block-tapping test [CBT: (87)] was used to assess short- and long-term topographic memory. Specifically, the WalCT measures the memory of short paths in the navigational vista space, which is, according to Wolbers and Wiener (88), the portion of the navigational space that can be seen from a single location or with little exploratory movements.

It was set up in a wide room of the Department of Human Neuroscience. It was composed of nine green squares (30 × 30 cm) that were placed on a carpet on the floor, reproducing the same standard positions as used in the CBT (see Figure 1A). In this test, the subject had to walk and reach different locations. The experimenter illustrated the sequence by walking on the carpet and stopping on each square for 2 s. Then, the subject had to repeat the same sequence as the experimenter by walking and stopping on the squares included in the sequence.

Following standard procedures (51, 65, 66), participants performed three different tasks, assessing:

1. *Topographic short-term memory (TSTM)*, in which a square span was obtained, that is, the number of squares in the longest sequence of squares that a child was able to repeat in the correct order immediately after the presentation.
  During the task, the number of squares to be reproduced gradually increased in length (from a 2-square sequences to a 9-square sequences). Children were required to reproduce the square sequences shown by the examiner by actually stepping on the green squares included in the sequence previously shown by the examiner.
2. *Topographic learning (TL)*, in which participants were asked to learn a fixed supra-span sequence (a path), which was calculated by considering the square span of the child + 2 squares according to standard procedures (5, 89). In each trial, after the examiner presented the sequence, the child was invited to walk on the carpet to reproduce it, stepping out of the carpet when he/she had finished. In each trial, the number of correct black squares reproduced in the sequence was calculated as the final score. During the task no feedback about the correctness of performance was provided. The learning criterion (indicating that learning was achieved) corresponded to three consecutive correct reproductions in a row of the sequence; in case the child did not reach the learning criterion the sequence was repeated for a maximum of 18 trials (5, 89).
3. *Topographic delayed recall (TDR)* of the supra-span sequence, in which 5 min later (the child and examiner spent this time out of the room where the WalCT was placed), participants were required to show the learned sequence. The examiner did not remind the child of the sequence and the child had to reproduce what he/she remembered of the long previously learnt sequence.
  At the end of the TDR task, the children had to use a felt-tip marker to retrace the pathway they had learned on the outline map of the WalCT (Figure 1B).

The whole protocol was administered in a single session on ~30 min at the Department of Human Neuroscience.

##### Neuropsychological Testing Administered to ADHD Children

For the aim of this study, descriptions and scores of the “attention and executive function” and “memory and learning” domains of NEPSY II (90) and the working memory indexes of WISC IV (91, 92) are reported.

#### NEPSY II

##### Attention and Executive Functions

In NEPSY II (90), the subtests administered to the children with ADHD in the domain “attention and executive function” were as follows:

**Animal sorting (AS):** assessing the ability to formulate basic concepts, to sort those concepts into categories, and to shift from one concept to another.
**Auditory attention (AA):** assessing selective auditory attention and the ability to sustain it (vigilance).
**Visual attention (VA):** assessing the ability to sustain selective visual attention.
**Response set (RS):** the first task assesses the ability to shift and maintain a new and complex set involving both the inhibition of previously learned responses and correctly responding to matching or contrasting stimuli. In the second task, the child listens to a series of words and touches the appropriate circle when he/she hears a target word.
**Design fluency (DS):** assessing the child's ability to generate unique designs by connecting up to five dots, presented in two arrays: structured and random.
**Inhibition (IN):** assessing the ability to inhibit automatic responses in favor of novel responses and the ability to switch between response types (90).

The scores of the children with ADHD on the “attention and executive function” subtests of NEPSY II (90) are reported in Table A1.

#### Memory and Learning

In NEPSY II (90) the subtests administered to children with ADHD in the “memory and learning” domain are as follows:

**List memory (LM):** assessing verbal learning and memory, rate of learning, and the role of interference in recall for verbal material.
**Memory for designs (MFD):** assessing the spatial memory for novel visual material.
**Memory for faces (MFF):** assessing the ability to encode facial features, as well as face discrimination and recognition.
**Memory for names (MFN):** assessing the ability to learn the names of children over three trials.
**Narrative memory (NM):** assessing the memory for organized verbal material under free recall, cued recall, and recognition conditions.
**Sentence repetition (SR):** assessing the ability to repeat sentences of increasing complexity and length.
**Word list interference (WLI):** assessing the verbal working memory, repetition, and word recall following interference (90).

The scores of the children with ADHD on the “memory and learning” subtests of NEPSY II (90) are reported in Table A2.

##### WISC IV

*Working memory indexes* This index is a measure of an individual's ability to hold verbal information in short-term memory and to manipulate that information. It consists of two subtests:

**Digit span (DS):** The examiner reads a series of numbers and the child has to repeat them. Then, the examiner again reads a series of numbers but this time the child is required to say them back in reverse order.
**Letter number sequencing (LNS):** The examiner reads a series of letters and numbers and the child is required to repeat them back with the letters in alphabetical order and the numbers in numerical order (The WISC IV and WAIS IV Subtests, Weiss, data, 2003). Table A3 reports the working memory indexes (WMI) of WISC IV (91, 92) of children with ADHD.

### Statistical Analysis

To compare the performances of the two groups (ADHD-C vs. TD group) in the WalCT, we used the g formula of Hedges and Olkin (93), which can be interpreted with the same rules as Cohen's d (94). Indeed, Hedges' g and Cohen's d are extremely similar except when the sample size is small, in this case, Hedges' g outperforms Cohen's d (95). Values for Hedges' g that range from 0.20 to 0.49 are reported as small effect sizes. Values that range from 0.50 to 0.79 are reported as moderate effect sizes, and values of 0.80 or more are reported as large effect sizes. Rosenthal (96) added a classification of very large, defined as being equivalent to or >1.30. In addition to the effect size, we computed the 95% confidence interval (CI) for the effect size, which is usually interpreted as the range of values that encompass the population, or the true value, estimated by a certain statistic with a given probability (97–99). According to Nakagawa and Cuthill (100), when we have a mean difference of 28 with 95% CI = −1 to 59, the result is not statistically significant (at an α level of 0.05) because the CI includes zero, while another mean difference of 28 with 95% CI = 9–49 is statistically significant because the CI does not include zero. Differences in the number of perseverative errors, committed by the two groups during WalCT, were evaluated by the Chi-square (Chi^2^) test. Zero-order correlation was conducted to measure the strength and direction of association that exists between the ADHD-C group's performances in the WalCT and the performances in NEPSY II (90) (attention, executive function, and memory and learning domains) and in the WISC-IV (working memory index) (91, 92).

### Statistical Power of the Sample

To our current knowledge, this is the first study evaluating topographic memory in children with ADHD-C of a school age (ranging from 8 to 10 years) thus we conducted a *post-hoc* power analysis (i.e., OBSERVED power) based on the effect size, sample size, and parameter estimate from the data set, which found the following observed power for the variables considered: TSTM (0.93), TL (0.67), RN (0.76), OM (0.26), WalCT perseverative errors (0.88).

### Preliminary Results

Regarding the control variables of age and Raven score, the two groups were balanced for age but not for Raven score with a moderate Hedges' g (ADHD-C group: M = 24.67, SD = 5.67; TD group: M = 27.67, SD = 2.44; g: −0.67; CI = −1.40 to 0.07).

However, in examining the 95% CI for the effect size, for the Raven score, a mean difference of −3 with a 95% CI = −1.40 to 0.07 was not statistically significant because the CI included zero. Furthermore, the two groups were balanced for gender; the ADHD group was composed of three girls and 12 boys and the TD group consisted of five girls and 10 boys [${\text{Chi}}_{\left(1\right)}^{2}$ = 0.682; *p* > 0.05]. All children of both groups were of Caucasian origin and came from families with middle-class socio economic status.

## Results

### NEPSY II

#### Attention and Executive Function Domain

In our sample, the majority of children with ADHD showed below-average performances in the attention and executive function domain of NEPSY II (90), in particular 67% of children performed below average on the animal sorting (AS) subtest, 80% on the auditory attention (AA) subtest, 40% on the visual attention (VA) subtest, 80% on the responses et (RS) subtest, 60% on the design fluency (DF) subtest, and 74% on the inhibition (IN) subtest (Table A1).

### Memory and Learning Domain

In the memory and learning domain of NEPSY II (90), 60% of children performed below average on the list memory (LM) subtest, 47% on the design fluency (DF) subtest, 40% on the memory for designs (MFD) subtest, 33% on the memory for faces (MFF) subtest, 20% on the memory for names (MFN) subtest, 47% on the narrative memory (NM) subtest, 47% on the sentence repetition (SR) subtest, and 47% on the word list interference (WLI) subtest (Table A2).

### WISC- IV

Below average performances on the working memory index of the WISC-IV (91, 92) were reported by 40% of children with ADHD, 27% on the digit span (DS) subtest and 33% on letter number sequencing (LNS) (Table A3).

### Walking Corsi Test (WalCT)

By comparing the ADHD group with the TD group, it emerged that the ADHD group had significantly lower performance in several tasks. In particular, the Hedges' g ranged from 0.50 to 1.67 (i.e., from medium effect to very large effect) on the topographic short-term memory (TSTM) task, topographic learning (TL) task, repetition number task during the TL task, and the WalCT outline map task (Table 2); however, in examining the 95% CI for the effect size, for the WalCT outline map task, a mean difference of 23.34 with a 95% CI = −1.22 to 0.23 was not statistically significant because the CI included zero (Table 2). All children of both groups correctly performed the topographic delayed recall (TDR) task without errors, so this score was not inserted into the table. The skewness and kurtosis statistics appeared to be very dependent on the sample size. Smaller sample sizes can give results that are very misleading. The statistics for skewness and kurtosis simply did not provide any useful information beyond that already given by the measures of location and dispersion (101).

**Table 2:** Hedges' g-comparison between groups: diagnosis by the WalCT.

	**ADHD-C (*****n*** **=** **15)**				**TD (*****n*** **=** **15)**				**Means comparisons between groups**				
**Variables**	**M**	**SD**	**Skewness**	**Kurtosis**	**M**	**SD**	**Skewness**	**Kurtosis**	**Diff. means**	**Df**	Hedges' g^*^	**95%CI Hedges' g**	
TSTM	3.53	0.83	0.31	−0.23	4.93	0.80	0.13	−1.35	−1.40	28	**−1.67**	−2.50	−0.84
TL	89.89	6.24	−0.55	−0.57	95.13	3.80	−0.83	−0.36	−5.24	28	**−0.99**	−1.75	−0.23
RN	9.13	4.69	0.58	−0.89	5.07	1.49	0.92	−0.20	4.06	28	**1.14**	0.36	1.91
OM	43.95	48.00	0.37	−2.05	67.30	43.65	−0.74	−1.38	−23.34	28	−0.50	−1.22	0.23

* *For the interpretation of Hedges' g: > 0.20 is a small effect; > 0.50 is a medium effect; > 0.80 is a large effect. Rosenthal (96) added a classification of “very large” defined as being equivalent to or >1.30. Values in bold are significant*.

For the perseverative errors in the WalCT, the Chi^2^ test showed significant differences between the two groups with respect to the distribution of the levels of the variables [${\text{Chi}}_{\left(1\right)}^{2}$ = 8.88, *p* = 0.003]. In particular, the ADHD group committed a significantly higher number of perseverative errors than the TD group in the WalCT (Table 3).

**Table 3:** Perseverative errors (PE) in the WaLCT ADHD-C vs. TD.

	**Actual**	**Expected**	Adjusted residual^*^
**ADHD-C (*****n*** **=** **15)**			
Error count = 0	5	9	−3
Error count > 0	10	6	3
**TD (*****n*** **=** **15)**			
Error count = 0	9	13	3
Error count > 0	6	2	−3

* *The adjusted residuals are the raw residuals (or the difference between the observed counts and expected counts) divided by an estimate of the standard error. Chi (1) = 8.88, p = 0.003*.

Zero-order correlations showed a positive correlation between the topographic short-term memory (TSTM) task and auditory attention (AA) task (*r* = 0.55, *p* = 0.03), and the memory of design task (*r* = 0.67, *p* = 0.01) of NEPSY II, and the digit span task (*r* = 0.54, *p* = 0.04) of WISC-IV (Table 4). Furthermore, a correlation between delayed recall and response set (RS) (*r* = −0.66, *p* = 0.01) of NEPSY II was found (Table 4).

**Table 4:** Significant zero-order correlations between ADHD-C's performances on WalCT and NEPSY II and on WalCT and WISC-IV.

		**AA**	**RS**	**MFD**	**DS**
TST	*r*	0.55	0.19	0.67	0.54
	*p*	**0.03**	0.49	**0.01**	**0.04**
TDR	*r*	−0.22	−0.66	0.07	−0.05
	*p*	0.42	**0.01**	0.8	0.87

*r, Pearson correlation; p =Sig. (2-tailed) TSTM, topographic short-term memory; TDR, topographic delayed recall; AA, auditory attention; RS, response set; MFD, memory for designs; DS, digit span. Values in bold are significant*.

## Discussion

To our knowledge, the current study is the first to assess the topographic memory in children with a diagnosis of ADHD-C using the WalCT (4, 51, 65, 66), testing the subject's ability to get oriented while really walking and moving through the environment. We found that the presence of an ADHD-C diagnosis negatively affected topographic memory performances in the WalCT. In regard to the first hypothesis of this work, we found an impairment in the topographic short-term memory in children with ADHD-C. This finding is in line with previous studies on working memory and visuospatial short-term memory in children with ADHD (e.g., (35, 102)). Kofler et al. (76) found that impaired working memory in children with ADHD determined consistent difficulties in anticipating, planning, enacting, and maintaining goal-directed actions, all fundamental abilities for a successful navigation. Zero-order correlations were conducted to measure the association between the ADHD-C group's performances in the WalCT and the performances in NEPSY II (90) and in WISC-IV (91, 92), confirmed this finding. Indeed, we found a positive correlation between the ADHD-C group's performances on TSTM and on digit span of WISC-IV (working memory index). During the digit span task, the child had to repeat a dictated series of digits forwards and another series backwards. The forward condition assessed span capacity, and the backward condition evaluated the ability to manipulate information in working memory (103). Rosenthal et al. (96) compared the performances on forward digit recall and on backward digit recall in children with ADHD, finding that the ADHD-predominantly inattentive group was able to recall significantly more digits backward than the ADHD-combined type group, showing a specific deficit in working memory. They also found an association between working memory and cortical thickness of the left medial temporal lobe in a sample of children with ADHD during the backward digit span subtest of WISC-IV (104). A recent study (105) found that human spatial navigation training is associated with changes in cortical thickness. Participants exhibited large improvements in navigation performance after 4 months of training, and these improvements were partly maintained 4 months after the end of training. In according to neural plasticity, younger adults showed higher performance than older adults. Considering this evidence, it would be interesting to assess if spatial navigational training in children with ADHD-C might increase cortical thickness, and in turn improve working memory ability. Taking into account that for pre-school children with TD a navigational training program fostered the acquisition of survey representation that is typically developed much later (106).

Furthermore, our finding showed that TSTM appears to be correlated with auditory attention (AA). It assessed the selective auditory attention and the ability to maintain concentrated attention over prolonged periods of time (vigilance or sustained attention). Children with ADHD-C could show a lower TSTM than TD because they could not sustain their concentration during the presentation of the sequences by the examiner. So, they were able to repeat only the first squares presented. To support of this hypothesis, Tucha et al. (107) assessed attention functioning in children with ADHD—predominantly hyperactive-impulsive type and children with ADHD—combined type, and showed that in comparison to healthy participants, they were impaired in measures of vigilance, divided attention, selective attention, and flexibility. Also, Pasini et al. (42), found that an ADHD-C and ADHD-I group showed an impairment in divided attention, response inhibition (prepotent response and interference control), and phonological and visual-object working memory.

Eventually, TSTM appears to be correlated with the memory for designs (MFD) task of NEPSY II that assessed spatial memory for novel visual material. In memory for designs, the child is shown a grid with four to ten designs on a page, which is then removed impeding the child from seeing the material to remember. The child then selects the designs from a set of cards and places the cards on a grid in the same location as previously shown (108).

A low score on MFD may suggest difficulty with route memorization for details, with location of visual stimuli details in the two-dimensional space and with learning for visuospatial information. Although there are few studies using tests to assess visuo-spatial working memory (VSWM) in children with ADHD-C, our findings are consistent with Westerberg et al. (35), who showed that in a specific sample of boys with ADHD-C and with no comorbidity (like our sample expect for the gender), VSWM is an important component of ADHD symptomatology. Also (37) investigated visuo-spatial and verbal WM performance in children with combined-type ADHD and their matched controls, finding that children with ADHD-C had deficits, relative to controls, on tasks designed to assess visuo-spatial WM. It is important to highlight that a delay in cortical maturation in children with ADHD has been reported by several authors [e.g., (62–64)] which showed a marked delay in children with ADHD in attaining peak thickness in particular in the prefrontal regions important for attention, planning, and navigational abilities: the median age of the attained peak thickness of cortical points for the ADHD group was 10.5 years, which was significantly later than the median age of 7.5 years for TD children.

Furthermore, children with ADHD-C performed significantly worse than controls in topographic learning (TL), they made more mistakes in executing the square-sequence path and needed a significantly higher number of repetitions to learn the path than children with typical development.

It is known that the maintenance of attention and cognitive flexibility play an important role in learning (47, 49, 50). Cognitive flexibility (CF) allows us to rapidly adapt our thoughts and behaviors in response to changing environmental demands and goals (109, 110), and CF is related specifically to working memory and inhibitory control (111). Cognitive flexibility plays a relevant role in learning and in complex problem solving. It allows us to select the strategy we need to adapt to different situations, capturing information from the environment, and responding in flexible ways by adjusting our behavior to the changes and demands of the situation. Perseverative errors committed by participants on executive function tasks such as the Wisconsin card sort test [WCST, (112)] are said to reflect cognitive inflexibility. An interesting finding of this work, that could have important consequences for clinical practice, is the fact that children with ADHD-C committed more perseverative errors during the topographic learning (TL) tasks than the TD children, showing more cognitive inflexibility. It is recognized that children with ADHD have difficulty in changing their responses even when the feedback suggests that their response is ineffective or maladaptive (75, 82, 113–115). Ahmadi et al. (82) investigated the differences between ADHD subtypes in terms of executive function profile, finding that children with the ADHD combined type showed more perseverative responses and perseverative errors than children with the ADHD predominantly inattentive type and TD children. In the study conducted by Lawrence et al. (113), children's performance on both neuropsychological and real-life measures of executive function and processing speed were compared, finding that problems in goal-directed behavior during real-life measures (number of deviations from designated route) were related to problems in set-shifting on the WCST (perseverative responding). Vallesi et al. (115) demonstrated that drug-naïve ADHD children were less able than TD children to switch from quick to accurate decision making, when required by the task demands, showing a deficit in the flexible regulation of strategic behavior. According to Fuster (116) and Barkley (33), perseverative errors in children with ADHD could be due to an interaction between behavioral inhibition and working memory; they fail to hold in mind information on the success of their response immediately preceding trials (retrospection), which then feeds forward to influence or even stop immediate future responses. On this topic, a growing body of research has focused on neural correlates of error detection reflected in the event-related potential (ERP). In particular, two ERP components seem to be involved in error processing: error-related negativity (ERN) reflecting a “monitoring system” to detect errors, and error positivity (Pe) reflecting a “remedial action system” to compensate for errors (117). Children with ADHD seem to have no compromises in early error monitoring processes related to error detection, whereas they show abnormal response strategy adjustments (117). In accordance with these findings, recent research demonstrated that it is very difficult to learn from our mistakes. Monfardini et al. (118) administered the same task in rhesus macaques and humans in which they could see that two items concealed a reward (a coin for humans, a candy for macaques). The authors found that choice-induced preference strongly affected individual learning. The monkeys and humans performed much more poorly after an initial negative choice than after an initial positive choice, indeed, poor learning from errors due to over-valuation of personal choices is among the decision-making biases shared by humans and animals.

Furthermore, Robaey et al. (119) based on their finding suggested a different strategy to help children with ADHD symptoms better orientate in space. They found a significant interaction between the presence of ADHD symptoms and learning strategy in virtual navigation tasks suggesting that children with ADHD symptoms primarily rely on caudate nucleus-dependent response learning strategies at the expense of hippocampus-dependent spatial strategies. They suggest that promoting a response strategy (counting, using anon-spatial systematic pattern of open and closed pathways, etc.) could help them to be better oriented when introducing them to a new spatial environment.

Unlike previous studies (120, 121), we did not find differences in the delayed recall between the two groups. From our results, it would seem that once the children have learned the path, they have no difficulty in keeping it in their memory and repeating it after 5 min. Although several long-term memory (LTM) tests used this interval of time or even shorter (e.g., Rey Figure Memory Recall) and in accordance with Miller's and Atkinson and Shiffrin's definition of short-term memory (STM), 5 min are a short time of delay and it is possible that some differences may emerge with longer time intervals. Future studies should also take into account different long-term intervals of time. Based on our findings, ADHD-C seemed to be associated with impaired initial topographic learning, probably due in particular to selective auditory attention and to the ability to maintain attention over prolonged periods of time (vigilance or sustained attention), to poor working memory and spatial memory for novel visual material, and perseverative behavior (cognitive inflexibility), whereas long-term retention of the learned topographical material was not compromised. This finding is consistent with previous studies that found that children with ADHD tend to display intact LTM if the material is encoded successfully (122, 123), suggesting that if children with ADHD have encoded information into long-term memory, they have no difficulties in retaining and retrieving that information (124).

We did not find significant differences between the two groups on the WalCT outline map task. Generally, all children of both groups showed difficulties in reproducing the learned path on the map. This finding is consistent with previous studies that showed how some aspects of human navigation are acquired later mainly because the neural bases underlying different processes have different maturational rates. Indeed, the neural structures that allow path integration and reorientation are already fully developed in toddlers, whereas more complex navigational skills require the activity of neural areas (such as the prefrontal cortex, PFC) which present a prolonged development, continuing into adolescence (51, 54).

## Conclusions

Based on our findings it would seem that children with ADHD-C have a poor topographic span and a specific impairment in learning a new path, but when it is acquired, they have no difficulty in keeping it in their memory and repeating it after 5 min. In fact, children with ADHD-C presented significantly worse performances than controls in the topographic short-term memory (TSTM) and topographic learning (TL) tasks but not in topographic delayed recall (TDR). These impairments are probably due to the difficulty of maintaining their attention over prolonged periods of time (sustained attention), a spatial memory for novel visual material, poor working memory and visuo-spatial working memory, and perseverative behaviors. In regard to perseverative behaviors, children with ADHD-C tend to repeat the committed errors even if feedback suggests that their response is ineffective or maladaptive. Based on these results and on the conducted studies by Monfardini et al. (118), it would be interesting to design topographic training for children with ADHD-C based on the observation of errors of other subjects, in order to assess if they are able to overcome the bias linked to the choice-induced preference and commit fewer perseverative errors, learning the new path faster.

This study presents some limitations: the sample size does not allow for generalizations about findings and for such a reason we could not investigate the presence of individual differences (such as gender) on topographic memory in our sample, although previous studies found no gender differences in children aged 4–11 years on WalCT performance (4, 51). We followed strict criteria of inclusion in the sample recruitment: diagnosis of ADHD-C (DSM-5), age 8–10, IQ ≥85, an average score on Raven's colored progressive matrices (84, 85), no previous treatment, no other diagnosis of comorbidity, no primary visual or hearing impairments, and no other neurological or organic disease. These criteria led to the recruitment of a small sample of children with ADHD-C. In particular, it is very difficult to find a child with pure ADHD, because the presence of a comorbid disorder in neurodevelopmental disorders, and in particular, in ADHD children is more the rule than the exception (125). Also, the average age of diagnosis for ADHD is usually 6–8 years, so it is difficult to recruit children with pure ADHD-C who received their first diagnosis after the age of 8. As for the control group, we conducted a formal interview with parents and teachers to obtain information about the children but the influence of emotional variables (i.e., anxiety) was not considered in the neurocognitive performance.

Further studies should compare different subtypes of ADHD [predominantly inattentive ADHD (ADHD/I) vs. hyperactive-impulsive subtype (ADHD/HI) vs. ADHD-C] to evaluate if there are differences between these groups in topographic memory and to better understand the role of attention, hyperactivity, and impulsivity in navigational performances.

Eventually, several previous studies showed cortical maturation delay in ADHD children, particularly in prefrontal regions, relevant for the attention, planning, and navigational abilities that often disappear during adolescence [e.g., (61–64, 126–129)]. It would be interesting to assess further studies on topographic memory in older ADHD children compared with younger ADHD children in order to investigate whether part of the difficulties displayed by young ADHD-C children could be explained by a developmental delay that improves over time.

## Data Availability Statement

The original contributions presented in the study are included in the article/Supplementary Material, further inquiries can be directed to the corresponding author/s.

## Ethics Statement

The studies involving human participants were reviewed and approved by Ethics Committee Of Policlinico Umberto I, Rome, Italy (Protocol number:416/16). Written informed consent to participate in this study was provided by the participants' legal guardian/next of kin. Written informed consent was obtained from the individual(s), and minor(s)' legal guardian/next of kin, for the publication of any potentially identifiable images or data included in this article.

## Author Contributions

MV recorded clinical data. NF conducted the research and wrote the manuscript with LP. SR and DA analyzed clinical data and conducted the statistical analysis. VG, GN MR, and CG coordinated and supervised the work and critically reviewed the manuscript. VB revised the English language editing. All authors have read and approved the final manuscript.

## Conflict of Interest

The authors declare that the research was conducted in the absence of any commercial or financial relationships that could be construed as a potential conflict of interest.
