# Supplementary material for: Link Between Topographic Memory and the Combined Presentation of ADHD (ADHD-C): A Pilot Study
Source: Front Psychiatry. 2021 Jun 17;12:647243. doi: 10.3389/fpsyt.2021.647243 (PMC8245696; doi:10.3389/fpsyt.2021.647243)
Supplement: Supplementary file 1 [file Data_Sheet_1.docx]

**Appendix A**

| **Table A1: Attention and Executive Functions Domain of the NEPSY-II (Korkman et al. 2011)** | | | | | | | |
| --- | --- | --- | --- | --- | --- | --- | --- |
| Subject | Gender  (Age) | Animal Sorting (AS) | Auditory Attention (AA) | Visual Attention (VA) | Response Set (RS) | Design Fluency (DF) | Inhibition  (IN) |
| 1 | M (9) | 7 | 26°–50° | 5 | 26°–50° | 5 | 26°–50° |
| 2 | M (9) | 11 | 26°–50° | 7 | >75° | 14 | 11°–25° |
| 3 | M (9) | 10 | 2°–5° | 1 | 2°–5° | 4 | 2°–5° |
| 4 | M (8) | 6 | 2°–5° | 9 | 6°–10° | 7 | 11°–25° |
| 5 | M (8) | 9 | 11°–25° | 8 | 26°–50° | 7 | 11°–25° |
| 6 | M (9) | 9 | 2°–5° | 12 | 2°–5° | 7 | 26°–50° |
| 7 | M (8) | 5 | 11°–25° | 1 | 11°–25° | 11 | 2°–5° |
| 8 | M (9) | 7 | 2°–5° | 5 | 11°–25° | 8 | 2°–5° |
| 9 | M (9) | 12 | 26°–50° | 9 | 6°–10° | 5 | 26°–50° |
| 10 | M (10) | 7 | 11°–25° | 9 | 11°–25° | 6 | 11°–25° |
| 11 | M (9) | 6 | 11°–25° | 9 | 2°–5° | 8 | 11°–25° |
| 12 | M (8) | 6 | 6°–10° | 14 | 11°–25° | 16 | 11°–25° |
| 13 | F (10) | 5 | <2° | 5 | 2°–5° | 5 | 11°–25° |
| 14 | F (8) | 7 | <2° | 10 | 11°–25° | 4 | 51°–75° |
| 15 | F (8) | 7 | 6°–10° | 8 | 11°–25° | 8 | 11°-25° |

Note: 13–19= >75 (Above Expected Level); 8–12 = 26–75 (At Expected Level); 6–7 = 11-25 (Borderline); 4–5 = 3–10 (Below Expected Level); 1–3 = ≤2 (Well-Below Expected Level) (Miller, 2007)

| **Table A2: Memory and Learning Domain of the NEPSY-II (Korkman et al. 2011)** | | | | | | | | |
| --- | --- | --- | --- | --- | --- | --- | --- | --- |
| Subject | Gender (Age) | List Memory (LM) | Memory for Designs (MD) | Memory for Faces (MF) | Memory for Names (MFN) | Narrative Memory  (NM) | Sentence Repetition  (SR) | Word List Interference  (WLI) |
| 1 | M (9) | 10 | 13 | 7 | 12 | 11 | 12 | 11 |
| 2 | M (9) | 5 | 8 | 4 | 8 | 7 | 8 | 6 |
| 3 | M (9) | 2 | 6 | 8 | 10 | 6 | 3 | 4 |
| 4 | M (8) | 7 | 7 | 8 | 10 | 11 | 12 | 9 |
| 5 | M (8) | 13 | 13 | 8 | 15 | 13 | 15 | 14 |
| 6 | M (9) | 7 | 10 | 7 | 7 | 7 | 8 | 12 |
| 7 | M (8) | 11 | 13 | 12 | 11 | 13 | 9 | 9 |
| 8 | M (9) | 6 | 10 | 6 | 6 | 10 | 5 | 5 |
| 9 | M (9) | 14 | 10 | 10 | 10 | 11 | 11 | 7 |
| 10 | M (10) | 5 | 10 | 8 | 8 | 6 | 6 | 5 |
| 11 | M (9) | 8 | 7 | 11 | 10 | 7 | 5 | 8 |
| 12 | M (8) | 6 | 6 | 8 | 14 | 10 | 11 | 8 |
| 13 | F (10) | 6 | 5 | 8 | 8 | 6 | 6 | 5 |
| 14 | F (8) | 8 | 5 | 14 | 10 | 3 | 7 | 11 |
| 15 | F (8) | 6 | 8 | 6 | 6 | 8 | 6 | 5 |

Note: 13–19= >75 (Above Expected Level); 8–12 = 26–75 (At Expected Level); 6 –7 = 11–25 (Borderline); 4–5 = 3–10 (Below Expected Level); 1–3 = ≤2 (Well-Below Expected Level) (Miller, 2007)

| **Table A3: Working Memory Index of WISC-IV (Wechsler, 2003; Orsini et al. 2012)** | | | | |
| --- | --- | --- | --- | --- |
| Subjects | Gender (Age) | Digit Span (DS) | Letter-Number Sequencing (LNS) |  |
| 1 | M (9) | 13 | 9 | 106 |
| 2 | M (9) | 9 | 5 | 82 |
| 3 | M (9) | 9 | 3 | 76 |
| 4 | M (8) | 8 | 7 | 85 |
| 5 | M (8) | 9 | 10 | 97 |
| 6 | M (9) | 13 | 13 | 118 |
| 7 | M (8) | 12 | 7 | 97 |
| 8 | M (9) | 6 | 8 | 82 |
| 9 | M (9) | 10 | 6 | 88 |
| 10 | M (10) | 9 | 10 | 97 |
| 11 | M (9) | 8 | 9 | 91 |
| 12 | M (8) | 8 | 8 | 88 |
| 13 | F (10) | 4 | 10 | 82 |
| 14 | F (8) | 4 | 8 | 79 |
| 15 | F (8) | 6 | 8 | 82 |

Note: Weakness Point <85; Normal = 85–115; Strong point <11

Supplementary files

• Wechsler Intelligence Scale for Children, fourth edition (Wechsler, 2003) or Weschler Preschool and Primary Scale of Intelligence, third edition (Wechsler, 2013) in order to exclude children and adolescents with intellectual disabilities and borderline functioning;

• DDE 2 (Batteria per la valutazione della Dislessia e della Disortografia evolutiva 2- *Battery to assess Developmental Dyslexia and Dysorthography 2*: Sartori et al., 2007) in order to evaluate dyslexia and dysorthography;

• Prove di Lettura MT per la Scuola Elementare-2 (*Reading Tests MT for primary School-2* Cornoldi & Colpo, 1998) and Nuove prove di lettura MT per la scuola media inferiore (*New Reading Test MT for lower middle school*: Cornoldi & Colpo, 1995) to evaluate dyslexia and reading comprehension;

• Batteria Discalculia Evolutiva (BDE) (*Battery for Developmental Dyscalculia*: Biancardi & Nicoletti, 2004) and Batteria AC-MT (Battery A-MT. Cornoldi et al., 2002) to evaluate numeracy and dyscalculia;

• Developmental Test of Visual-Motor Integration (VMI) (Beery, 1997), in order to evaluate the growth of visuospatial and visuomotor skills in childhood;

• Prova per la valutazione della scrittura in età evolutiva (BHK) (Task to assess the writing in developmental age: Di Brina & Rossini, 2011) to evaluate dysgraphia;

• NEPSY-II (Korkman et al., 2011) to evaluate the development of neuropsychological function.

Emotional and behavioral profile:

• Big Five Questionnaire Children, self-report version (Barbaranelli et al., 1998);

• Children’s Depression Inventory (CDI) (Kovacs, 1986);

• Multidimensional Anxiety Scale for Children (MASC) (March et al., 1997);

• Thematic Apperception Test (TAT) (Murray, 1943);

• Children’s Apperception Test (CAT) (Bellak & Bellak, 1949);

• Graphic Tests: Draw-a-person Test, Kinetic Family Drawing, Invented Family Drawing.

Clinical survey and interviews for parents and teachers:

• Child Behavior Checklist (CBCL 6-18) and Teacher Report Form (TRF) (Achenbach & Rescorla, 2001);

• Conners’ Parent and Teacher Rating Scales (revised version) (Conners, 1989);

• Kiddie Schedule for Affective Disorders and Schizophrenia Present and Lifetime version (K SADS PL), adapted by Kaufman et al. (1997, 2000, 2004).
